# Treatment of Refractory Metastatic Renal Cell Carcinoma

**DOI:** 10.3390/cancers14205005

**Published:** 2022-10-13

**Authors:** Joseph A. Vento, Brian I. Rini

**Affiliations:** Vanderbilt-Ingram Cancer Center, Vanderbilt University Medical Center, Nashville, TN 37232, USA

**Keywords:** metastatic renal cell carcinoma, refractory therapies, immune checkpoint inhibition, VEGFR tyrosine kinase inhibitors, new RCC agents

## Abstract

**Simple Summary:**

The treatment of metastatic renal cell carcinoma continues to rapidly evolve, with various combinations of immune checkpoint inhibitors and targeted tyrosine kinase inhibitors improving outcomes in the first-line setting. However, a significant subset of patients fail to respond to these therapies, and many patients eventually progress, necessitating effective therapeutic options in the treatment-refractory setting. Here, we review the treatment of refractory renal cell carcinoma, including the current standard of care, ongoing trials with the potential to alter current paradigms, and considerations for non-clear cell histologies.

**Abstract:**

First-line treatment for metastatic renal cell carcinoma (mRCC) rapidly shifted in recent years with the advent of combination therapies, including immune checkpoint inhibitor (ICI) doublets and combinations of an ICI with a vascular endothelial growth factor receptor (VEGFR) targeted tyrosine kinase inhibitor (TKI). Despite improvements in overall survival and many durable responses, there exists a significant number of patients who fail to respond to these agents, and many patients eventually progress. Given the rapid changes in the front-line setting, it is essential to understand treatment options in refractory mRCC. Here, we review the evidence behind current options for later-line therapies, often involving additional VEGFR-TKIs alone or in combination with mammalian target of rapamycin (mTOR) targeted agents, as well as situations where consideration of immunotherapy rechallenge may be appropriate. Additionally, we describe ongoing clinical trials examining concurrent ICI and TKI in the refractory setting, as well as those studying novel agents, such as targeted drug–antibody conjugates and hypoxia inducible factor 2α (HIF-2α) inhibitors. Finally, we review considerations for non-clear cell histologies in the refractory setting and mechanisms of resistance in mRCC.

## 1. Introduction

There are 79,000 cases of kidney and renal cancer diagnosed annually in the United States, with renal cell carcinoma (RCC) making up over 80% of adult cases [1,2]. A third of RCC cases present as locally advanced or metastatic, and a significant percentage of tumors managed with localized therapies recur, often in distant metastatic sites [3,4]. In recent years, the addition of immune checkpoint inhibitor (ICI) doublets and ICI agents, given concurrently with vascular endothelial growth factor receptor (VEGFR) targeted tyrosine kinase inhibitors (TKIs), rapidly changed the first-line treatment standard for non-resectable or metastatic renal cell carcinoma (mRCC) through repeated demonstration of improved overall survival relative to sunitinib in the first-line setting [5,6,7,8]. However, a significant number of patients fail to respond to these treatments, with objective response rates (ORRs) across the landmark trials of these combinations of therapies ranging from 42% to 71%. Many patients eventually progress, with progression-free survival (PFS) in these trials ranging from 11.6 to 23.9 months. In addition, many patients experience toxicities on these therapies, and 22% to 37.2% require discontinuation of one or both drugs due to toxicities. 

With these significant changes in first-line therapies, the landscape for management of future-line therapies must also evolve. The primary therapeutic options in the refractory setting for mRCC include VEGFR TKIs, ICIs (monotherapy or ICI doublet), and agents targeting the mammalian target of rapamycin (mTOR) pathway, often in combination with a TKI. The choice of second- and later-line therapies depends on the first-line therapy received, availability of clinical trials, comorbidities of patients, and patient preference. In this paper, we will review each class of refractory therapy, including important trials for specific therapeutic agents in each class, and a framework for navigation sequential treatment of refractory mRCC based on prior therapies. Additionally, we will review several promising agents undergoing clinic trials or which are in early phase studies, and conclude with a discussion on evidence specific to non-clear cell mRCC and mechanisms of resistance in mRCC. 

## 2. Results

Here, we review the different classes and specific treatment agents in refractory mRCC, and when to think about using them in sequential therapy. We primarily focus on clear cell histology, comprising the large majority of metastatic RCC cases, and conclude with evidence for non-clear cell histologies. While ICI and TKI combination therapy or ICI doublet therapy are currently the preferred first-line therapy options, we also consider scenarios in which the first line of therapy involves TKI monotherapy or ICI monotherapy.

### 2.1. VEGFR TKIs in the Refractory Setting 

VEGFR TKIs inhibit a variety of pathways involved in tumorigenesis of mRCC and provide an effective refractory mRCC treatment option regardless of initial systemic therapy. The selection of the specific agent largely depends on the initial treatment received. Data supporting the activity of VEGFR TKI as subsequent therapies for mRCC is summarized in Table 1. Note, prospective data demonstrating activity of TKI monotherapy after prior ICI (with or without prior TKI) exists only for cabozantinib (phase II and a small subset of phase III patients), tivozanib (phase III), and axitinib (phase II). More robust prospective trials show activity of this class of agents after progression on prior TKI monotherapy, including cabozantinib (phase III), lenvatinib with everolimus (phase III), tivozanib (phase III), and axitinib (phase III). Sunitinib and pazopanib have robust data in the first-line setting but no recent prospective evaluation in later-line settings, limiting understanding of their usefulness after prior ICI and TKI therapies [9,10]. Prospective data for use of TKIs use following ICI and TKI combination therapy exists primarily in subgroups of larger trials not examining this situation as a primary outcome; thus, we also reviewed several retrospective studies, demonstrating encouraging results for a variety of TKI agents after combination first-line therapy.

The mechanism of specific VEGFR TKIs approved in mRCC provides additional insight into their effectiveness in the refractory setting, their side effect profiles, and biology to consider when deciding on subsequent therapies. Clear cell RCC frequently upregulates the VEGF pathway to propagate angiogenesis via inactivation of the von Hippel–Lindau (VHL) tumor suppressor gene. While all VEGFR TKIs inhibit this pathway, they differ in the scope of additional tyrosine kinases targeted. For example, cabozantinib also inhibits c-MET and AXL, both known to be upregulated in RCC and to predispose to metastatic spread, contributing to its effectiveness in the front-line and refractory setting but also to its wide range of side effects in these populations. Tivozanib, on the other hand, targets very specifically the VEGFR pathway, which grants a more favorable side-effect profile. Interestingly, the phase III trial demonstrating response to tivozanib in the refractory setting included patients already treated with broader targeting TKIs, highlighting the need to further explore how sequential treatment with TKIs of varying ranges of kinase targets influences the therapeutic effectiveness and resistance mechanisms of mRCC.

#### 2.1.1. Cabozantinib

Cabozantinib is a TKI with multiple targets, including *VEGFR*, *c-MET*, *AXL*, and *RET*, all known to be active in tumorigenesis and propagation of mRCC. Cabozantinib is an effective option after any prior therapy not already including cabozantinib and has become the default standard of care in the refractory setting.

The phase III METEOR trial first examined cabozantinib in the TKI-refractory setting versus everolimus, and showed an improvement in overall survival (OS) and PFS in this population, including 5% who had received prior nivolumab therapy [11]. Since then, this agent progressed into the first-line setting in combination with nivolumab based on data of the CheckMate 9ER study showing improved OS and PFS relative to sunitinib [7].

Since the advent of ICI doublet and ICI/TKI combination therapy, limited prospective exists for cabozantinib in the refractory setting. The phase II BREAKPOINT trial showed a 43% ORR and 37% rate of stable disease in 48 patients, the majority of which (74%) had received prior ICI doublet therapy, with 17% having received prior ICI/TKI and 9% having received adjuvant ICI [12].

Furthermore, in a retrospective review of patients pretreated with ICI therapies, either alone or in combination with TKIs, cabozantinib demonstrated an ORR of 36% and median OS of 13.1 months and 55% OS at 12 months [13]. In the subgroup of patient pretreated with ICI and TKI combination therapy, the ORR was 28%, and in the subgroup pretreated with ICI monotherapy or ICI doublet therapy, the ORR was 42%. A total of 16.3% of patients discontinued therapy due to toxicities. In two separate retrospective cohorts looking at cabozantinib and other treatments after prior ICI therapy, cabozantinib had an ORR of 53.5% in one [14] and a combined partial response (PR) or stable disease (SD) rate of 53.2% in the other [15].

CaboPoint is a phase II trial (NCT03945773) that will provide prospective data for cabozantinib in the second-line setting after current standard-of-care first-line treatments [16]. It will evaluate ORR as the primary endpoint in patients receiving cabozantinib as second-line therapy in unresectable RCC after prior ICI doublet or ICI/TKI therapy. Each group will have an enrollment of 125 patients, and recruitment is ongoing.

#### 2.1.2. Tivozanib

Tivozanib is a selective *VEGFR* inhibitor with a long half-life, which is useful in refractory RCC, and is an agent with proven activity after multiple lines of prior therapy. Its narrower spectrum of kinase inhibitor portrays a favorable side-effect profile, especially in patients with extensive comorbidities or intolerable of other TKIs.

The TIVO-3 study provided phase III evidence for this drug as a third- or fourth-line of therapy through comparison to sorafenib, demonstrating improved PFS and ORR [17]. The tivozanib arm included 175 patients, with 45% of patients having received 2 VEGFR TKIs, 27% having received ICI/TKI combination therapy, and 28% receiving TKI and another agent (most often everolimus).

In the subgroup analysis by IMDC risk factor, tivozanib improved PFS for favorable- and intermediate-risk RCC, but not for poor-risk disease. There is some thought that poor-risk RCC is less susceptible to tivozanib due to being less driven by angiogenesis, the primary target of the selective VEGFR inhibition of this agent.

Recently updated PFS data from TIVO-3 demonstrate a small subgroup of patients with long-term progression-free survival on tivozanib (7.6% at 48 months), with no correlate (0%) in the sorafenib arm [18]. Additionally, recently updated OS data from TIVO-3 demonstrate a trend of improved OS with declining hazard ratios, with additional accrual of events (HR 0.89 at a mean of 22.8 months, with 280 events, vs. HR 0.99 at the original endpoint at a mean of 17.9 months, with 227 events) [19].

Tivozanib in combination with nivolumab is being evaluated in the phase III TiNivo-2 study versus tivozanib alone in patient with 1–2 lines of prior therapy, one of which must include an ICI [20]. This will be further discussed in the immune checkpoint inhibitor section of this review.

#### 2.1.3. Lenvatinib plus Everolimus

Lenvatinib is a TKI targeted at VEGFR, fibroblast growth factor receptor (FGFR), c-KIT, PDGFR, and RET. It is approved in combination with the pembrolizumab in the first-line setting based on the results of the 2021 CLEAR trial showing superiority in OS and PFS to sunitinib [8]. In the refractory setting, it is used in combination with mTOR inhibitor everolimus, and shows effectiveness after prior TKI therapy not including lenvatinib. Response to lenvatinib and everolimus after prior therapies may be due to expanded targeting of the mitogen-activated protein kinase (MAPK) and mTOR pathways.

A 2015 phase II study of lenvatinib plus everolimus examined its role in mRCC previously treated with a TKI, and found the combination of the two led to a longer PFS than everolimus alone [21]. No prospective data exist after prior ICI therapy. However, in a retrospective series of 55 patients treated with lenvatinib with or without everolimus who had all received both prior ICI and TKI therapies (though only 10% received the two concurrently), 21.8% of patients had an objective response and 63.8% had stable disease. The majority (83.6%) had already received cabozantinib. The median PFS was 6.2 months and OS was 12.1 months [22].

The phase Ib/II KEYNOTE-146 study looking at lenvatinib plus pembrolizumab includes patients in the refractory setting and will be discussed in more detail in the immune checkpoint inhibition section of this review [23].

#### 2.1.4. Other TKIs

The remaining VEGFR TKIs approved in mRCC include axitinib, sunitinib, pazopanib, and sorafenib. Of these, only axitinib has positive prospective data in the refractory setting, and only following therapies that are no longer standard mRCC treatments, with the exception of sunitinib. Sunitinib and pazopanib remain viable options in further-line settings, though they have only been evaluated prospectively in the first-line setting. Data on sorafenib in the refractory setting comes from its use as a comparator arm in trials with tivozanib and axitinib.

Axitinib improved PFS over sorafenib in the second-line setting in the 2013 AXIS trial, when TKI monotherapy with sunitinib was the standard first-line practice [24]. A phase II prospective study looking at personalized dose-adjusted axitinib after ICI therapy, though again prior to widespread first-line ICI and TKI dual combination therapy, did not meet the targeted PFS threshold, but found 45% of patients had an objective response in this setting [25].

In summary, beyond cabozantinib, tivozanib, lenvatinib with everolimus, and axitinib, there exist little prospective data to aid in selecting the optimal TKI.

### 2.2. ICI Alone or in Combination with Another ICI or TKI in the Refractory Setting

The use of immune checkpoint inhibitors in mRCC began in the refractory setting, with nivolumab in the 2015 Checkmate 025 study, and quickly progressed to all first-line therapies, including ICI as a part of a doublet. In the refractory setting, ICIs are most critical to consider in ICI-naïve patients; however, re-challenge with ICI agents alone or in combination presents an additional opportunity for consideration and the subject of multiple recent early phase trials. Relevant combinations and trials are summarized in Table 2. There are no validated biomarkers to aid in predicting which mRCC patients will respond to these therapies in any line of therapy.

#### 2.2.1. Nivolumab and Ipilimumab

Nivolumab is a programmed cell death protein 1 (PD-1) inhibitor that first demonstrated clinical response in the mRCC refractory setting after progression on a VEGFR TKI in the phase III Checkmate 025 trial, demonstrating improved OS compared to everolimus [26]. In any patient naïve to immune checkpoint inhibition without contraindications, it should be prioritized as a subsequent therapy. However, since incorporating ICIs into doublets in the first-line setting, it remains unclear if later-line nivolumab provides additional benefits, as limited prospective data examining this scenario exist.

Adding ipilimumab, another ICI targeting cytotoxic T-lymphocyte-associated protein 4 (CTLA4), to nivolumab monotherapy after lack of a clinical response to monotherapy demonstrates limited benefit as a salvage therapy. The HCRN GU16-260-Cohort A examined this scenario and found the ORR of the combination regimen in 35 patients that did not respond to monotherapy to be 11.4% [27]. In the OMNIVORE trial, only two of the 57 patients who received salvage ipilimumab converted to a PR [28]. In TITAN_ RCC, this salvage therapy was examined in both the first- and second-line setting after prior TKI, and found 3 of 16 patients to have a PR with ipilimumab boost in the first-line setting, and 2 of 10 patients to have a PR in the second-line setting [29].

The phase II FRACTION-RCC trial examined rechallenge with nivolumab with or without ipilimumab after prior ICI therapy, and found a 16% ORR [30]. This indicates some patients may respond to rechallenge or intensifying ICI therapy; however, no guidance exists as to which subset of patients may respond.

#### 2.2.2. ICI and TKI Dual Therapy

With the recent incorporation of ICI/ICI and ICI/TKI dual therapies in the first-line setting, a natural follow-up question is the effectiveness of ICI/TKI dual therapy in the refractory setting after prior combination therapies. Several early phase studies show encouraging results in this population, including the combinations of lenvatinib with pembrolizumab and tivozanib with nivolumab.

The KEYNOTE-146 trial is a phase Ib/II study that examined the efficacy of lenvatinib and pembrolizumab in combination in the second-line setting [23]. Of the 145 patients, 104 patients were pre-treated with ICI, including 39 patients who had received ipilimumab with nivolumab and 18 patients who had received ICI/TKI combination therapy. Subgroup analysis of these groups showed an ORR of 61.5% and 38.9%, respectively, highlighting the potential of this therapy even after standard-of-care first-line combination therapies.

The phase Ib/II TiNivo study looked at the combination of tivozanib and nivolumab in 25 patients, including 13 pre-treated patients, in the refractory setting and showed a 62% ORR in the pre-treated population [20]. The phase III TiNivo-2 (NCT04987203) will follow up on this study and examine the role of tivozanib with nivolumab versus tivozanib monotherapy in the refractory setting in patients who previously received 1–2 lines of therapy, including ICI [31]. The recruitment goal for this study is 326 patients; active enrollment is ongoing. The primary outcome will be PFS.

The phase Ib COSMIC-21 study of atezolizumab and cabozantinib demonstrated the efficacy of this combination, primarily in the first-line setting, in both clear and non-clear cell histologies [32], and the ongoing CONTACT-03 phase III trial (NCT04338269) plans to enroll 500 locally advanced or mRCC patients, and will examine PFS in this combination in the refractory setting immediately following prior ICI therapy [33].

In addition to early phase prospective data, one retrospective review reported on 52 patients who received ICI and TKI combination therapy in the second-line or later setting [34]. Prior to receiving this dual therapy in the later-line setting, 19.2% had received prior dual ICI therapy, 51.9% had received TKI and ICI sequentially, and 29% had only received TKI therapy. In the second-line setting, the study noted a 37.5% response rate, with ORR declining with each additional line of prior therapy. In the patients who received prior ICI dual therapy, the ORR was 50%, and in patients with prior TKI and ICI sequentially, the ORR was 20.8%.

### 2.3. mTOR Inhibitor Monotherapy in the Refractory Setting

Everolimus is an inhibitor of the mTOR pathway that is often overactive in mRCC, and remains an option for refractory RCC. Its primary use is in in combination with the TKI lenvatinib, as reported in the TKI review above. However, CheckMate02 showed a 5% overall response rate to everolimus monotherapy, and the METEOR trial showed an ORR of 3% to everolimus in the refractory setting after prior TKI therapy [26,35]. Similarly, temsirolimus, an intravenous mTOR inhibitor, has response rates of 5% in the refractory setting after prior TKI therapy [36]. One could consider these agents as monotherapy in the refractory setting after progression through a lenvatinib-containing earlier-line regimen, though this warrants a discussion with patients of the limited response rates of this agent as monotherapy versus the side-effect profile.

### 2.4. Novel and Early Phase Therapeutic Agents in the Refractory Setting

A number of early phase trials exhibit promise of new targeted agents in the treatment of refractory mRCC, summarized in Table 3.

#### 2.4.1. Belzutifan

Belzutifan is a hypoxia inducible factor-2α (HIF-2α) inhibitor now approved in patients with cancers associated with von Hippel–Lindau (VHL) disease. As the majority of sporadic RCC is also driven by mutations in VHL and the resulting upregulation of HIF-2α, inhibition on HIF-2α provides a promising target in mRCC. Ongoing trials examine the role of belzutifan in the refractory setting. In the recently presented phase I/II LITESPARK-001 3-year follow-up data, belzutifan showed ongoing efficacy in clear cell mRCC in patients who already progressed on one other line of therapy, including 71% having already received both TKI and ICI [37]. Notably, the PFS rate at 36 months was 34%, indicating the potential for durable response in these patients.

Additionally, a phase II trial of belzutifan in combination with cabozantinib in mRCC in the refractory setting demonstrated a high disease-control rate (complete response, partial response, or stable disease) of 92.7% [38]. The combination of HIF-2α with TKI will be further evaluated in an ongoing phase III study of belzutifan in combination with lenvatinib vs. cabozantinib in the refractory setting after prior ICI therapy [39].

#### 2.4.2. Telaglenastat + TKI or mTOR Therapy

Telaglenastat is a glutaminase inhibitor that showed promise in the phase II trial ENTRATA, in combination with everolimus compared to everolimus monotherapy, in extending PFS for refractory mRCC after multiple previous lines of therapy (100% received prior TKI and 88% received prior ICI) [40]. However, the more recent phase II CANTATA results compared telaglenastat in combination with cabozantinib vs. cabozantinib alone and found no difference in PFS [41]. Thus, additional trials with combinations of this therapy are needed to determine the activity of this agent in refractory mRCC.

#### 2.4.3. Batiraxcept plus TKI Therapy

Batiraxcept is a fusion protein of immunoglobulin G heavy chain with an extracellular region of human AXL, and functions in AXL inhibition, known to be upregulated via HIF2α signaling. Additionally, AXL is overexpressed in tumors with resistance to TKI therapies. Early phase Ib/II data in 26 patients indicated responsiveness to batiraxcept (AVB-S6-500) in combination with cabozantinib in patients with advanced clear cell renal cell carcinoma who had already received front-line treatment, with a 46% partial response rate [42]. In patients with an upregulated sAXL to GAS6 ratio, the ORR was 67%, indicating this as a potential predictor of response to this therapy. Additional data for this agent in other combinations and settings are anticipated.

#### 2.4.4. Histone Deacetylase Inhibitors

Histone deacetylase inhibitors (HDACi) present a potential therapeutic option in refractory mRCC, as histone deacetylases are often overactivated in mRCC and alter regulation of genes that contribute to tumorigenesis [43]. A 2011 phase II trial of the HDACi panobinostat failed to show any objective responses in 20 patients [44]. However, more recent early phase trials show encouraging activity when these agents are used in combination with other classes of medications known to be effective in mRCC. Vorinostat, another HDACi, in combination with the VEGF monoclonal antibody bevacizumab, demonstrated an ORR of 18% in refractory mRCC [45]. Further, phase 1 data from the combination of panobinostat with everolimus showed tolerable toxicities and a median PFS of 4.1 months in pre-treated mRCC patients [46]. Additional trials are needed to help elucidate the role of this class of therapy in refractory mRCC.

#### 2.4.5. Ongoing Early Phase Trials and Other Novel Therapies

A Phase II study of 14 patients with mRCC of the Lutetium-labeled anti-carbonic anhydrase IX (CAIX) monoclonal antibody girentuximab demonstrated 8 patients with stable disease and 1 with partial regression [47]. Prior treatments were allowed but the majority of patients were treatment naïve, with favorable-risk disease. Unfortunately, the majority of patients developed grade 3–4 myelosuppression.

The STARLITE 2 phase 2 study of nivolumab in combination with the 177 Lutetium-labeled CAIX monoclonal antibody girentuximab will further explore this therapy as a combination therapy with immunotherapy in mRCC patient previously treated with ICI [48]. Radiation may stimulate additional antigen presentation and enhance the efficacy of immunotherapy, hence the rationale for this combination.

Chimeric antigen receptor modified T cell (CAR-T) therapy already demonstrates encouraging results in hematologic malignancies, but limited data support its use in solid malignancies such as mRCC. A 2016 12-patient phase I/II study looked at CAIX-targeted CAR-T therapy in CAIX-expressing mRCC but found no benefit [49]. Another group examined the combination of sunitinib with CAR-T therapy targeted at CAIX in a mouse metastatic xenograft RCC model, demonstrating that while sunitinib or the CAIX CAR-T alone had limited anti-tumor effect, the combination decreased tumor burden and prolonged survival [50]. VEGF TKIs, such as sunitinib, are known to improve T-cell infiltration and cytokine response, as well as upregulate CAIX expression; this being the rationale behind combining the two agents. CRISPR Therapeutics is examining a CD70-directed CAR-T agent CTX130 in refractory RCC patients, with a phase 1 trial ongoing (NCT04438083) [51]. CD70 is a transmembrane protein within the tumor necrosis factor receptor family often expressed in clear cell RCC.

The recently published data on axitinib with and without anti-OX40 antibody (pF-04518600) investigated 59 patients in the refractory mRCC setting and found no improvement in PFS (13.1 vs. 8.5 months, HR 0.85, *p* = 0.61) for the addition of an anti-OX40 antibody [52]. Additionally, recently published phase II data examining the PARP inhibitor talazoparib and avelumab in VHL deficient clear cell RCC showed no objective tumor responses [53]. Volociximab, an antibody against the α_5_β_1_ integrin, known to be upregulated in tumor angiogenesis, presented another potential agent in refractory mRCC. In a phase II trial of 40 patients, it showed an 80% stable response rate, with four patients having a time to progression of greater than 14 months [54]. However, no randomized controlled studies of this agent have been done to date.

### 2.5. Refractory Non-Clear Cell RCC Treatment Options

Non-clear cell renal cell carcinoma (nccRCC) represents a heterogenous group of malignancies, making up 25% of all renal cell carcinoma cases [55]. Management of refractory metastatic nccRCC is a challenge given the variety in histology amongst these malignancies, with limited prospective data to guide treatment decisions in the refractory setting. The majority of the prospective studies already discussed included only clear cell histology or included only small subsets of nccRCC patients, and thus were unable to generate robust data.

In the first-line setting, management of metastatic nccRCC varies by histology, but often include similar therapies to metastatic ccRCC, such as anti-VEGF TKIs, ICIs, other targeted agents such as MET inhibitors, and more rarely cytotoxic chemotherapy. A detailed discussion on the selection for a specific subtype of nccRCC is beyond the scope of this paper. Refractory treatment is often extrapolated from clear cell data, despite the heterogeneity in histologies. Specific agents with prospective or informative retrospective data in the refractory settings are summarized in Table 4. Of note, there is very limited refractory-specific data, rather, many of these trials allowed for pre-treated patients to be included in the cohorts.

Cabozantinib represents an attractive option in the refractory setting, just as in metastatic clear cell RCC. A large retrospective cohort including a large number of pretreated patients showed a 27% ORR in this population [56]. Immunotherapy, particularly if not used in the first-line setting, represents another option in the refractory setting. Single-agent nivolumab in the CheckMate 374 trial showed an ORR of 13.6%, including 27% pre-treated patients [57]. Several phase II trials of immunotherapy combinations show a modest effect in cohorts of metastatic nccRCC that included pretreated patients [32,58,59,60]. Ongoing trials will shed further light on these agents’ effectiveness in the refractory setting. The remaining options for refractory metastatic nccRCC are extrapolated from clear cell RCC data, or from front-line metastatic nccRCC trials.

### 2.6. Mechanisms of Resistance in mRCC

The modern front-line landscape for mRCC treatments consists of ICI-based doublets, often including a VEGFR TKI, and the refractory treatment landscape frequently involves additional VEGFR TKIs alone or in combination with other agents. To better understand progression of mRCC while on these therapies, we briefly review the mechanisms of resistance to TKIs and ICIs in mRCC.

Development of resistance to VEGFR TKIs in mRCC involves upregulation of hypoxic survival pathways as tumors evolve to escape dependence on VEGF-driven angiogenesis [61]. Hypoxia not only encourages development of VEGF-independent angiogenesis pathways, but also results in additional effects, such as increased lysosomal degradation of TKIs. Agents such as belzutifan, which targets HIF2α, may be effective due to their ability to overcome these hypoxic escape pathways. Further, as *AXL* upregulation is one of the alternative VEGF-independent pathways for angiogenesis [62], agents such as batiraxcept appear promising in mRCC that is refractory to TKI therapy. Additional mechanisms of TKI resistance include recruitment of normal tissue vessels for blood supply and transition from an epithelial phenotype to a mesenchymal one, increasing the tumor’s ability to metastasize and decreasing susceptibility to TKI therapy.

Resistance to ICIs in mRCC emerge from changes in the tumor itself or changes in the tumor microenvironment. Tumor-specific resistance mechanisms include mutations in pathways such as the interferon gamma pathway, the MAPK pathway, and the PI3K/AKT/mTOR pathway [61]. Tumor microenvironment resistance mechanisms are of special interest in mRCC given it is one of the most T cell-infiltrated tumors, and the degree of T-effector gene expression along with other variables may even help predict upfront patient response to immunotherapy [63]. Immune mechanisms such as tumor associated macrophage infiltration, pro-inflammatory conditions, and escape to other immune checkpoints are several mechanisms of resistance to ICIs. For example, overexpression of another immune checkpoint, lymphocyte activation gene-3 (LAG-3), confers resistance to PD-1 inhibitors [64]. Immunotherapy agents targeting LAG-3 have shown promise in melanoma and are undergoing evaluation in mRCC [65].

## 3. Conclusions

The primary options for refractory mRCC in the era of front-line ICI combination therapy include VEGFR TKIs, mTOR-targeting agents in combination with TKI or as monotherapy, and additional novel agents.

Regarding TKIs, cabozantinib remains the default option for many patients who received ICI combination therapy not already including this agent in the front-line setting. Axitinib, lenvatinib in combination with everolimus, and tivozanib also have refractory-specific data supporting their use in the era of first-line ICI combination therapy. The usefulness of other TKIs, particularly sunitinib and pazopanib, is less clear given no refractory-specific prospective data for these agents. One must consider the tyrosine kinase targets and side-effect profile of these agents prior to initiation, though no clear data exists for sequencing TKI therapies. It is important to remember that refractory RCC options are not known to be curative, and so the tolerability of a given regimen is likely as important as clinical activity, with the choice of regimen adapted to each individual patient.

ICI monotherapy, particularly nivolumab, is most useful in the refractory setting for ICI-naïve patients. Several early phase prospective trials show the promise of ICI combination therapies after front-line ICI combination therapy, and ongoing phase III trials of tivozanib with nivolumab and atezolizumab with cabozantinib will clarify their role in the modern refractory landscape.

The new targeted agents highlighted in this review may prove efficacious refractory therapies in the coming years, and ongoing trials will provide insight into their usefulness in this space. Refractory non-clear cell mRCC is largely treated with similar agents as refractory clear cell mRCC; however, further non-clear cell-specific regimens are sorely needed.

## Figures and Tables

**Table 1:** VEGFR TKIs in the refractory mRCC setting, with the relevant trials.

Treatment	Study/Trial Design	N	Prior Therapies	Overall Survival	Objective Response Rate	Progression Free Survival or TTF *	Grade 3 or 4 Toxicity
Cabozantinib	Phase III vs. everolimus, METEOR	658 (330 vs. 328)	1+ TKI (5% prior ICI)	21.4 vs. 16.5 months (HR 0.66)	17% vs. 3%	7.4 vs. 3.9 months (HR 0.51)	71% vs. 60%
	Phase II control arm, CANTATA	223	TKI or dual ICI		28%	9.2 months	79%
	Phase II, BREAKPOINT	48	Adjuvant or first line ICI		43%	9.3 months	34%
	Retrospective Review, NCT03744585	86	ICI or ICI+TKI	13.1 months	36%	6.5 months (TTF)	15.1%
	Retrospective Review, Meet-Uro 7	79	ICI		53.2% (ORR or SD *)	7.6 months	40.5%
	Retrospective Review, NCT04353765	187	ICI	61.5% at 12 months	53.5%	6.2 months (TTF)	31.3% D/C * due to toxicities
Lenvatinib + Everolimus	Phase II vs. everolimus, NCT01136733	91 (51 vs. 50)	TKI	25.5 vs. 15.4 months (HR 0.51)	43% vs. 6% (RR 7.2)	14.6 vs. 5.5 months (HR 0.4)	71% vs. 50%
	Retrospective Review, PMID 33792094	55	ICI and TKI (80% 2+ TKIs)	12.1 months	21.8% (63.6% SD, 1 CR)	6.2 months	7.3% D/C due to toxicity
Tivozanib	Phase III vs. Sorafenib, TIVO-3	350 (175 vs. 175)	2+ including TKI	At 22.8 months, HR 0.89, CI 0.70-1.14	18% vs. 8%	5.6 vs. 3.9 months (HR 0.73)	11% vs. 10%
Axitinib	Phase III vs. Sorafenib, AXIS	723 (361 vs. 362)	Sunitinib or other **	20.1 vs. 19.2 months (HR 0.969)	8.3 vs. 5.7 months (HR 0.66)	23% vs. 12%	17% vs. 12% HTN *
	Phase II NCT02579811	40	ICI (70% prior TKI)	8.8 months		45%	60% HTN

* TTF—time to treatment failure; D/C—discontinue; SD—stable disease; HTN—hypertension. ** Cytokines, bevacizumab with interferon, or temsirolimus.

**Table 2:** ICI and ICI combination therapy in refractory mRCC.

Treatment	Study/Trial Design	N	Prior Therapies	Overall Survival	Objective Response Rate	Progression Free Survival	Grade 3 or 4 Toxicity
Nivolumab	Phase III vs. everolimus, Checkmate 025	821 (406 vs. 397)	1 or 2 TKI	25.0 vs. 19.6 months (HR 0.73)	25% vs. 5% (OR * 5.98)	4.6 vs. 4.4 months (HR 0.88)	19% vs. 37%
Nivolumab + Ipilimumab	Phase II, FRACTION-RCC	46	ICI (80.4% prior TKI)		15.2%		28.3%
Lenvatinib + pembrolizumab	Phase 1b/II study, KEYNOTE-146	145	ICI +/− TKI (104) TKI only (16)	Not reached 30.3 months	62.5% 52.9%	12.2 months 11.8 months	64% (pooled), 19% D/C * due to toxicity
Tivozanib + Nivolumab	TiNivo, Phase 1b/II study	25	13 pre-treated (unspecified) 12 first line		62% 50% (1 CR)	18.9 months (pooled)	80%, 32% D/C due to toxicity
ICI + TKI	Retrospective review, Cancer Med, 2022	85	33 first line 16 s line 16 third line 20 ≥ fourth line		56.7% 37.5% 21.4% 21%	15.2 months 14.2 months 10.1 months 6.8 months	27% (second line or beyond)

* OR—odds ratio; D/C—discontinued.

**Table 3:** Early phase trials in refractory mRCC.

Treatment	Mechanism of Action	Study/Trial Design	N	Prior Therapies	Objective Response Rate	Progression Free Survival	Overall Survival	Grade 3 or 4 Toxicity
Belzutifan Belzutifan + cabozantinib	HIF-2α inhibitor	Phase I/II LITESPARK-001	55	1+ therapies (81% ICI, 92% TKI)	25%	14.5 months		Anemia (76%), fatigue (71%)
		Phase II, NCT03634540	53	1-2 therapies	22%	16.8 months	95% at 6 months	HTN * (22.4%), Anemia (11.3%)
Telaglenastat + Everolimus Telaglenastat + Cabozantinib	Glutaminase inhibitor	Phase II vs. everolimus, ENTRATA	69	2+ therapies, at least one TKI	PR * or SD * in 58.7% vs. 47.8%	3.8 vs. 1.9 months (HR 0.64)	14.4 vs. 9.7 months (HR 0.8)	74% vs. 61%
	Glutaminase inhibitor	Phase II vs. cabozantinib, CANTATA	444	1-2 therapies, including TKI or dual ICI	31% vs. 28%	9.2 vs. 9.3 months (HR 0.94)		71 vs. 79%
Batiraxcept + cabozantinib	Fusion protein, IgG with portion of *AXL*	Phase Ib/II, NCT04300140	26	1+ therapy including ICI	46%	79% at 6 months		15%
Vorinostat + bevacizumab	Histone deacetylase inhibitor	Phase I/II single arm	36	1-2 therapies	18%	5.7 months	13.9 months	Fatigue (9%), TCP * (6%)

* PR—partial response; SD—stable disease; HTN—hypertension; TCP—thrombocytopenia.

**Table 4:** Trials in refractory non-clear cell mRCC

Treatment	Study/Trial Design	Histology	N	Prior Therapies	Overall Survival	Objective Response Rate	Progression Free Survival	Grade 3 or 4 Toxicity
Nivolumab	Phase IIIb/IV, CheckMate 374	Non-clear cell subgroup	44	34.1% + lines of therapy	52.8% at 12 months	13.6%	2.2 months	13.6%
Cabozantinib + Nivolumab Cabozantinib + Atezolizumab Cabozantinib	Phase II, NCT03635892	Non-clear cell only	47	0-1 therapies with no ICI	28 months	47.5%	12.5 months	32%
	Phase Ib/II, COSMIC-021	Non-clear cell subgroup	32	22% pre-treated		31%	9.5 months	38%
	Retrospective Review, [56]	Non-clear cell only	112	35% TKI, 32% ICI/TKI, 11% ICI	7.0 months	27%	51% at 12 months	17% (7% D/C * due to toxicity)
Bevacizumab+ atezolizumab	Phase II, NCT02724878	Non-clear cell subgroup	42	48% prior therapy		26%		
Durvalumab + savolitinib	Phase II, CALYPSO	Papillary only	41	Prior treatment allowed		27%	3.3 months	36.6%

* D/C—discontinued.

## Data Availability

No new data were created or analyzed in this study. Data sharing is not applicable to this article.
